# Biomechanics of Running Indicates Endothermy in Bipedal Dinosaurs

**DOI:** 10.1371/journal.pone.0007783

**Published:** 2009-11-11

**Authors:** Herman Pontzer, Vivian Allen, John R. Hutchinson

**Affiliations:** 1 Department of Anthropology, Washington University, St. Louis, Missouri, United States of America; 2 Structure and Motion Laboratory, Department of Veterinary Basic Sciences, The Royal Veterinary College, London, United Kingdom; Raymond M. Alf Museum of Paleontology, United States of America

## Abstract

**Background:**

One of the great unresolved controversies in paleobiology is whether extinct dinosaurs were endothermic, ectothermic, or some combination thereof, and when endothermy first evolved in the lineage leading to birds. Although it is well established that high, sustained growth rates and, presumably, high activity levels are ancestral for dinosaurs and pterosaurs (clade Ornithodira), other independent lines of evidence for high metabolic rates, locomotor costs, or endothermy are needed. For example, some studies have suggested that, because large dinosaurs may have been homeothermic due to their size alone and could have had heat loss problems, ectothermy would be a more plausible metabolic strategy for such animals.

**Methodology/Principal Findings:**

Here we describe two new biomechanical approaches for reconstructing the metabolic rate of 14 extinct bipedal dinosauriforms during walking and running. These methods, well validated for extant animals, indicate that during walking and slow running the metabolic rate of at least the larger extinct dinosaurs exceeded the maximum aerobic capabilities of modern ectotherms, falling instead within the range of modern birds and mammals. Estimated metabolic rates for smaller dinosaurs are more ambiguous, but generally approach or exceed the ectotherm boundary.

**Conclusions/Significance:**

Our results support the hypothesis that endothermy was widespread in at least larger non-avian dinosaurs. It was plausibly ancestral for all dinosauriforms (perhaps Ornithodira), but this is perhaps more strongly indicated by high growth rates than by locomotor costs. The polarity of the evolution of endothermy indicates that rapid growth, insulation, erect postures, and perhaps aerobic power predated advanced “avian” lung structure and high locomotor costs.

## Introduction

The metabolic physiology of dinosaurs and their extinct relatives – whether they were ectothermic (“cold blooded”) like modern reptiles, or endothermic (“warm blooded”) like birds and mammals – is important for reconstructing their ecology, behavior, and fate [1]–[6]. Endothermic animals are better able to inhabit a wide range of climates and maintain higher levels of activity than ectotherms, and thus much of our understanding of dinosaur paleoecology and evolution hinges on this critical distinction. Evidence for competing views of dinosaur metabolic physiology has come from a range of sources, including the anatomy of ventilatory organs [7]–[9], the apparent absence of ossified respiratory turbinates [10], reconstructed posture, habitat, and ecology [2], [3], [11]–[13], analogies with extant taxa based on experimental studies [13], the presence of feathers or filamentous integument [14], [15], and especially bone histology and growth [16]–[20], with much recent work suggesting at least some degree of endothermy.

High growth rates and perhaps activity levels seem to have been ancestrally present in the clade Ornithodira (dinosaurs, pterosaurs, and all descendants of their most recent common ancestor) [21], [22], in addition to perhaps some form of potentially-insulative filamentous integument [15]. Thus these characteristics apparently predate the parallel evolution, in pterosaurs and saurischians, of advanced respiratory systems (e.g., expanded air sacs and one-way air flow; [7]–[9]). Many studies take these features to indicate high metabolic rates and thus endothermy (or at least intermediate stages in its evolution) in extinct dinosaurs and other ornithodirans [6]. The question remains whether endothermy evolved earlier in Ornithodira, or independently in Pterosauria and Saurischia [e.g., 19]. Some researchers remain unconvinced of endothermy even in non-ornithurine birds (e.g. [10], [16]) or tentatively favor more complex scenarios involving multiple origins of endothermy [12]. Independent evidence would help distinguish between these alternative hypotheses.

In modern mammals and birds, endothermy incurs an energy cost of maintaining a high basal metabolic rate, but provides a substantial advantage in aerobic capacity over ectothermic reptiles. Whereas endotherms and ectotherms are capable of similar peak power output during short bursts, the maximum aerobically sustained metabolic rate, also termed “maximum aerobic power” or VO_2_max, is an order of magnitude greater for endotherms [23]. Consequently, sustained locomotor activity, which incurs similar costs for both groups [24], is relatively infrequent and limited to moderate running speeds in extant ectotherms [1], [25]. While increased locomotor activity might not have provided the initial evolutionary advantage for endothermy [26], [27], sustained aerobic activity provides a clear distinction between modern endothermic and ectothermic vertebrates; the metabolic physiology of modern ectothermic reptiles cannot sustain the aerobic activity commonly seen in birds and mammals.

**Figure 1:**
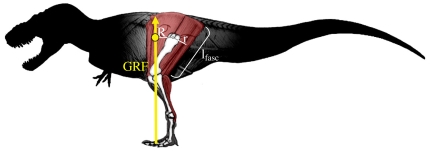
Schematic of extensor fascicle length (l_fasc_), the GRF vector moment arm (R; segmental gravitational, but not inertial, moments were also included but not shown here; see [**48**]), and the extensor (antigravity) muscle moment arm (r) for the hip joint. These parameters were calculated at midstance for the antigravity muscle groups at the hip, knee, and ankle, and combined with step length (estimated from hip height) to estimate the volume of muscle activated per meter travelled (V_musc_); see Methods. Joint angles and position of the center of mass (yellow circle) are taken from Hutchinson [40]. Adapted with permission from original artwork by Scott Hartman.

To assess the metabolic physiology of extinct dinosaurs and thereby test whether individual taxa fit endothermic or ectothermic models better, we compared estimated metabolic rates (oxygen consumption; mlO_2_ s^−1^) during walking and running in thirteen bipedal dinosaurs and one dinosauriform outgroup, *Marasuchus* (“*Lagosuchus*”; Sereno and Arcucci, 1994) to maximum aerobic power, VO_2_max (mlO_2_ s^−1^), for living endotherms and ectotherms. We used two recently developed methods linking locomotor anatomy to walking and running cost to estimate locomotor metabolic rates for dinosaurs (Fig. 1). Since walking and slow to moderate-speed running are aerobically sustainable in most modern amniotes [1], [25], [28], ectothermy in extinct species would be supported if their predicted locomotor costs at these moderate speeds fall within the aerobic capacity seen in living ectotherms. Alternatively, if the aerobic power needed for walking and slow running in these extinct species is predicted to exceed the maximum aerobic power for ectotherms, this would suggest these species were endothermic. We focused on bipedal species, because issues of weight distribution between fore- and hind limbs make biomechanical analysis of extinct quadrupeds more difficult and speculative.

## Results

**Figure 2:**
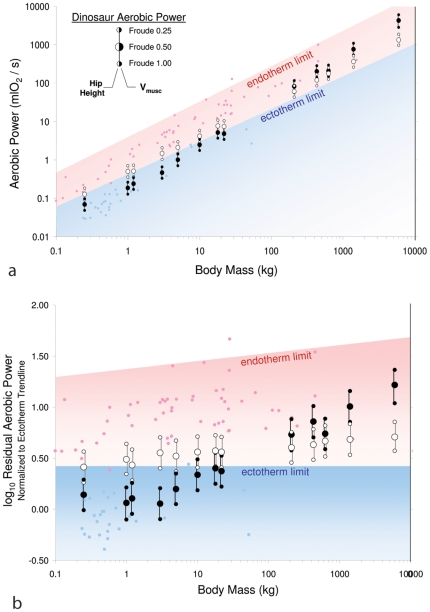
**A**. Locomotor power requirements for dinosauriforms (aeroic power, mlO_2_/s) plotted on a graph of maximum aerobic power (VO_2max_, mlO2/s), for extant endotherms (light red circles and shaded region) and ectotherms (blue circles and shaded region) versus body mass. Estimated rates of oxygen consumption for dinosauriforms are calculated using the two methods described in the text for walking (Froude 0.25), slow running (Froude 0.50), and moderate running (Froude 1.00) speeds (from left to right, *Archaeopteryx*, *Marasuchus*, *Microraptor*, *Compsognathus*, *Lesothosaurus*, *Heterodontosaurus*, *Coelophysis*, *Velociraptor*, *Gorgosaurus*, *Dilophosaurus*, *Plateosaurus*, *Allosaurus*, *Tyrannosaurus*). White symbols are estimates from hip height, black symbols are estimates from active muscle volume, V_musc_. The data points for *Coelophysis* and *Velociraptor* (both 20 kg) have been separated slightly for clarity. The upper limit of maximum aerobic power for modern ectotherms (i.e., the upper 95% confidence limit) is indicated by the upper boundary of the blue region; the upper limit for modern endotherms is indicated by the upper boundary of the red region. **B**. A similar plot as in A showing log_10_ residuals from the ectotherm trendline.

Surprisingly, the estimated locomotor metabolic rates for many dinosauriforms, especially larger taxa in our sample, consistently exceeded the 95% confidence interval for maximum aerobic power, VO_2_ max, seen in extant ectotherms (Figure 2). Locomotor power requirements (mlO_2_ s^−1^) estimated from hip height exceeded ectothermic capabilities at moderate running speeds (Fr 1.0) for all species, and at a slow run (Fr 0.50) for all but the smallest species (*Archaeopteryx*). Even during walking (Fr 0.25), the required metabolic output for the five largest species (a juvenile *Gorgosaurus*, *Dilophosaurus*, *Plateosaurus*, *Allosaurus*, and *Tyrannosaurus*) exceeded the range of aerobic capacity seen in extant ectotherms (Figure 2). Similarly, locomotor cost estimates based on active muscle volume, V_musc_, exceeded ectothermic capabilities at all walking and running speeds for the five largest species, and at moderate running speeds in the small, presumably active bipeds, *Heterodontosaurus*, *Compsognathus* and *Velociraptor*. Only the smallest species, *Archaeopteryx*, had estimated locomotor metabolic rates that fell within or near the range of VO_2_max seen in modern ectotherms, for all but the fastest speeds using both hip height and V_musc_ approaches (Figure 2; Table 1).

**Table 1:** Anatomical parameters, V_musc_, and locomotor costs estimated for thirteen extinct dinosauriforms.

						Locomotor Cost, VO_2_								
		Cost of Transport	Slow Walk (Fr 0.25)			Slow Run (Fr 0.50)			Moderate Run (Fr 1.0)		
Species	Mass kg	Hip Height cm	V_musc_ cm^3^ kg^−1^ m^−1^	Hip Height mlO_2_ kg^−1^ m^−1^	V_musc_ mlO_2_ kg^−1^ m^−1^	Speed m s^−1^	Hip mlO_2_ s^−1^	V_musc_ mlO_2_ s^−1^	Speed m s^−1^	Hip mlO_2_ s^−1^	V_musc_ mlO_2_ s^−1^	Speed m s^−1^	Hip mlO_2_ s^−1^	V_musc_ mlO_2_ s^−1^
*Archaeopteryx*	0.25	13	99.5	0.623	0.351	0.6	0.09	0.05	0.8	0.12	0.07	1.1	**0.18**	0.10
*Marasuchus*	1	14	55.5	0.595	0.223	0.6	0.35	0.13	0.8	**0.49**	0.18	1.2	**0.70**	0.26
*Microraptor*	1.2	24	41.2	0.385	0.181	0.8	0.35	0.17	1.1	**0.50**	0.24	1.5	**0.71**	0.33
*Compsognathus*	3	15	51.7	0.558	0.212	0.6	1.02	0.39	0.9	**1.44**	0.55	1.2	**2.03**	0.77
*Lesothosaurus*	5	26	40.7	0.367	0.180	0.8	1.46	0.72	1.1	**2.07**	1.02	1.6	**2.93**	1.44
*Heterodontosaurus*	10	27	39.1	0.355	0.175	0.8	2.89	1.43	1.2	**4.08**	2.02	1.6	**5.78**	**2.85**
*Coelophysis*	20	44	37.1	0.244	0.170	1.0	5.06	3.52	1.5	**7.16**	4.98	2.1	**10.12**	**7.04**
*Velociraptor*	20	42	44.8	0.253	0.192	1.0	5.13	3.89	1.4	**7.25**	**5.51**	2.0	**10.3**	**7.79**
*Gorgosaurus*	210	100	37.4	0.130	0.170	1.6	**42.6**	**56.0**	2.2	**60.2**	**79.2**	3.1	**85.2**	**112**
*Dilophosaurus*	430	116	44.5	0.116	0.191	1.7	**83.8**	**139**	2.4	**118**	**196**	3.4	**168**	**277**
*Plateosaurus*	630	110	27.5	0.120	0.142	1.6	**124**	**146**	2.3	**176**	**207**	3.3	**249**	**293**
*Allosaurus*	1400	149	47.3	0.095	0.199	1.9	**255**	**533**	2.7	**360**	**754**	3.8	**510**	**1066**
*Tyrannosaurus*	6000	264	44.1	0.061	0.190	2.5	**936**	**2898**	3.6	**1324**	**4099**	5.1	**1872**	**5797**

Cost estimates in bold are above the 95% confidence interval for ectotherm maximum aerobic power (VO_2_max) and thus more consistent with endothermy. See text and Figure 2 for details. The *Gorgosaurus* specimen is a juvenile. Hip heights, body masses, and anatomical dimensions are from [40] except *Marasuchus*, *Microraptor*, *Lesothosaurus*, and *Heterodontosaurus*; data for these latter species were estimated from whole-body digital models following [69; detailed descriptions of these models are unpublished, in preparation by Allen et al.]; and *Plateosaurus*, “slim model” from [70]. As the specific values have minimal effects on our analyses (Text S1) we do not consider potential error in body mass here. Specimens used for detailed measurements of the latter five taxa (especially segment lengths and moment arms) were a cast of the holotype of *Marasuchus* (“*Lagosuchus*;” BMNH R14101), the holotype of *Microraptor gui* (IVPP 13352; 3D sculpture by Jason Brougham, AMNH; based on specimen measurements), *Lesothosaurus* (BMNH RUB 17), *Heterodontosaurus* (UCMP 129614, cast of holotype), and *Plateosaurus* (GPIT 1).

Differences between hip height- and V_musc_-based estimates of dinosauriform locomotor cost highlight the different assumptions underlying each method. The reconstructed posture used to estimate V_musc_ employs more extended joints than expected for the smallest animals in our analysis [29]. More crouched poses, like those of similarly-sized extant species [29], would result in lower effective mechanical advantage (EMA) for the muscles, and hence produce higher estimates of locomotor cost [30]. This suggests that hip-height based estimates, which assume that EMA values for small dinosauriforms are similar to similarly-sized modern vertebrates, are likely more accurate for the small species in our dataset. Notably, hip height-based estimates consistently placed slow and moderate running costs for the smaller dinosauriforms within the endothermic range of aerobic output (Table 1, Figure 2). Conversely, the hip height approach assumes extremely extended limb postures for the largest dinosaurs, like those of walking elephants and other similarly-sized extant vertebrates. Such extended, “columnar” limb postures are incommensurate with the joint morphology and probable limb configuration of these species [5], [31], suggesting that the V_musc_ approach, which provides higher estimates of cost, is likely more accurate for the largest dinosaurs in our sample.

**Figure 3:**
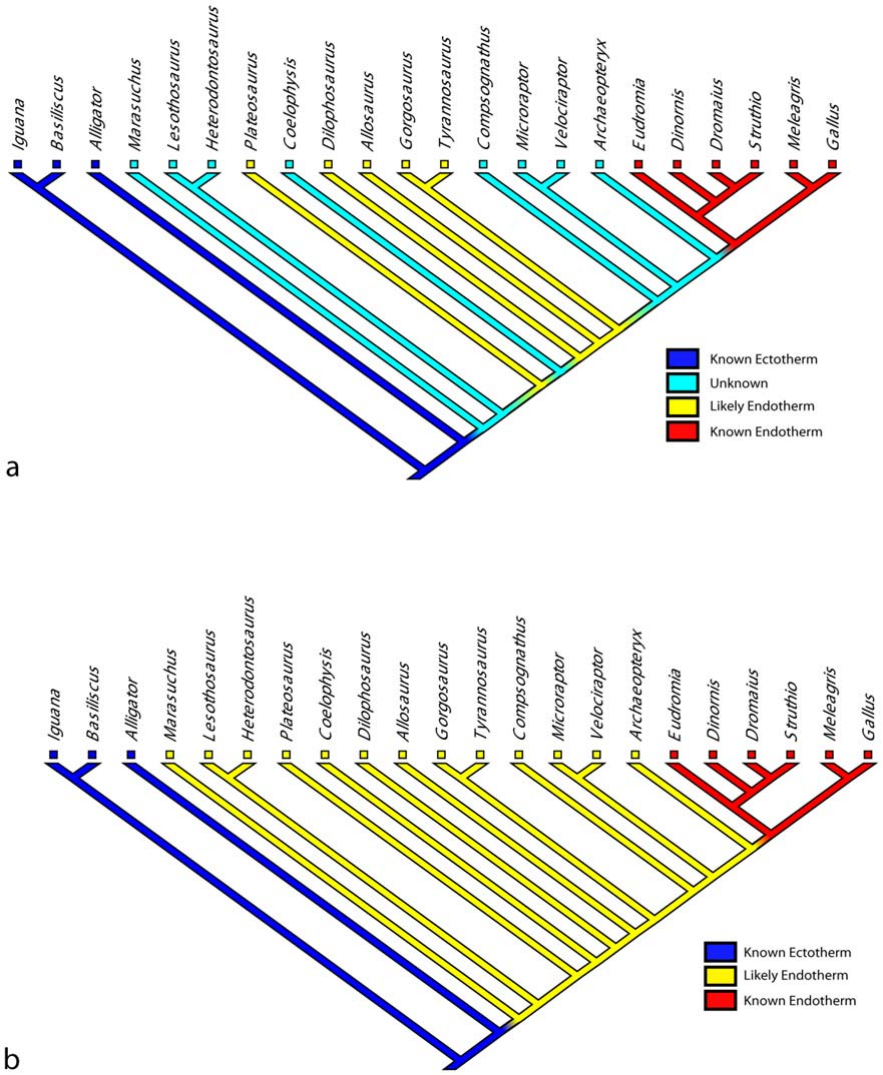
Evolution of locomotor cost and endothermy in Archosauria. **A**. Estimates from our V_musc_-based method for slow walking (Fr 0.25) used to reconstruct the evolution of endothermy. Substantial size-related homoplasy is shown. **B**. Estimates from our hip height-based method for moderate running (Fr 1.0) as a less conservative alternative to Fig. 2A. Endothermy is estimated as ancestral for at least Dinosauriformes.

Phylogenetic analysis of our data supports the hypothesis that an endothermic level of metabolism was needed to power at least slow running gaits in all Dinosauriformes (Figure 3). If a conservative approach is taken, using only locomotor cost estimates for slow walking, for which our two methods identify the same set of taxa as endothermic, then the hypothesis that endothermy arose independently at least three times (in Sauropodomorpha, Tetanurae and Neornithes) and was lost once (in Coelurosauria) is favored. This is at odds with insulatory and histological evidence for endothermy at least in coelurosaurs (14,15,18,19,20,22), but would correspond to some degree of coevolution of advanced ventilator structure, large body size and endothermy. An alternative, less conservative approach, using locomotor cost estimates for moderate speed running from our simple hip height-based method, supports the hypothesis that endothermic physiology and aerobic power were ancestral for dinosauriforms.

## Discussion

These results strongly suggest endothermy for larger (>20 kg) bipedal dinosaurs, because other explanations require physiological adaptations or locomotor limitations unseen in living terrestrial vertebrates. For example, it might be proposed that extinct dinosauriforms were ectothermic, but were able to achieve a greater VO_2_max than modern reptiles. While the respiratory physiology of dinosaurs has been the subject of much debate [1]–[8], [17], [19], the latter proposal would require an aerobic scope (VO_2_max/Resting Metabolic Rate) for the five largest species in our sample of 30–90x resting rate, far exceeding the maximum aerobic scope in our sample of modern ectotherms (14.5) and even greater than the aerobic scope of most mammals (mean 12.9±11.0 S.D.; max 61.9; see Table S2). Alternatively, it is possible that extinct dinosaurs/dinosauriforms did not engage in sustained locomotion, and instead used anaerobic metabolism to fuel short locomotor bouts. However, while intermittent locomotion is often used by modern lizards [32], this would suggest severely limited locomotor performance relative to living reptiles because even walking would need to be fueled anaerobically (Figure 2). Given the high post-exercise metabolic costs incurred by intermittent locomotion [32], utilizing an ectothermic “run now, pay later” strategy with such a high cost of transport would require problematically long, inactive recovery periods, particularly in the largest species. Finally, it could be argued that, by extrapolating the modern ectotherm aerobic power limit to the body size of large theropods, our approach underestimates the aerobic limits for large reptiles. However, while caution must always be used when extrapolating beyond the size range of comparative data, we see no evidence that relationship between aerobic capacity and mass changes at large body sizes among ectotherms or among endotherms.

Our results for smaller taxa are more ambiguous. The smallest species in our sample are estimated to exceed ectotherm aerobic capacities only at moderate running speeds (Fr = 1.0) and then only marginally (Figure 2). Thus, it is possible that these species were ectotherms that did not engage in sustained moderate or fast running. However, given the active lifestyles suggested for these species in other analyses [6], [33], [34] it is quite plausible that our conservative approach did not examine costs for sufficiently high speeds. This underscores an important limitation of our analysis. Because both endotherms and ectotherms are capable of producing low levels of aerobic power (i.e., below the upper power limit for ectotherms), species that require low aerobic power to fuel walking and moderate running speeds could be *either* endothermic or ectothermic. If the aerobic demands at these speeds can be met with either endothermic or ectothermic physiology, our approach cannot distinguish which physiology is more likely. Thus, while species that exceed ectothermic capabilities during slow and moderate locomotion may be reliably classed as endothermic, species that do not exceed ectothermic capabilities at these low and moderate speeds cannot be classed exclusively as ectotherms.

Further work on habitual speeds for the smaller species in our dataset would enable us to adjust the range of speeds modeled to better reflect those commonly used, and would improve the utility of this approach for those species. The limb poses of small dinosauromorphs also warrant further investigation. If the relatively extended postures input for our V_musc_-based estimates of cost are correct, this may indicate that relatively upright postures were a strategy that enabled small ectothermic dinosaurs to sustain moderate running speeds.

Large dinosaurs have sometimes been argued to be the most likely to have been ectothermic, based on “inertial homeothermy,” heat loss problems, and other constraints including ecological/energetic ones [3], [13], [35], [36]. Our study provides independent results that support the reconstruction of endothermy in large dinosaurs; indeed our method most clearly supports this inference for larger taxa. Likewise, as growth rates and other factors plausibly correlated with endothermy are reconstructed as having relatively different patterns and constraints in smaller- and larger-bodied taxa (e.g. [12], [19], [20], [35], [37], methods must quantitatively test for size-dependent influences on such factors. Our method does account for size effects, using a hip-height-based approach for smaller taxa and a muscle volume-based approach for larger taxa.

Sensitivity analyses (Text S1) indicate that our estimates of dinosaur locomotor cost are robust, especially for larger taxa. For example, for *Tyrannosaurus*, increasing hip height to reflect maximum joint extension only decreases hip height-based estimates of cost by 1.3% (Text S1). V_musc_-based estimates are similarly robust, with the high rates of muscle activation, and hence locomotor cost that are expected for the range of plausible musculoskeletal anatomy and poses in these species. Even for slow running (Fr = 0.5), estimated aerobic power in the large dinosaurs is 3 to 15 standard errors above the ectotherm VO_2_max trendline, or 100–500% above the upper bound for ectotherm aerobic power. In contrast, two-fold changes of the key unknown parameters (especially estimated body mass, moment arms, or l_fasc_), or moving the center of mass posteriorly to be coincident with the hip joint, result in a maximal total reduction of only 65% in estimated locomotor cost, still well above the ectotherm range for aerobic capacity (Text S1). Further, the V_musc_ approach works for species with known aerobic capacities: it correctly places the extinct moa (*Dinornis*) and moderate running speeds (Fr 0.5) for extant birds (*Gallus* and *Eudromia*) in the endotherm range, and walking and slow running in *Iguana*, *Basiliscus*, and *Alligator* in the ectotherm range for VO_2_max (Figure S1).

Our findings concur with multiple lines of independent evidence from bone histology [18], [19], [22] and cardiorespiratory anatomy [7], [8] indicating high growth rates and activity levels, if not endothermy, in Dinosauriformes. As locomotor costs were somewhat high for all dinosauriforms, this is consistent with the hypothesis that endothermy was ancestral for the entire clade. As pterosaurian outgroups had advanced bone histology [19], [21] and respiratory anatomy [38] as well as long muscular hindlimbs similar to those of dinosauriforms (although bipedalism/quadrupedalism remains controversial), the most parsimonious hypothesis is that endothermy first evolved in the shared common ancestor of Pterosauromorpha and Dinosauromorpha, in the clade Ornithodira. Since pterosaur anatomy, posture and gait remain highly controversial, and basal pterosauromorph taxa are quite small in body size, we considered it too speculative and ultimately ambiguous to apply our methods to pterosauromorphs, but predict that if such models could justifiably be done, our results would be further strengthened. Likewise, our analysis does not include quadrupedal dinosaurs, and therefore excludes many of the largest dinosaurs, such as sauropods. Analyses of the postures and weight distribution for these large quadrupeds are needed to test whether the high aerobic power requirements for bipedal forms seen here were common among large dinosauromorphs.

Our methodology adds a new, repeatable line of evidence that is explicitly and quantitatively linked with well-demonstrated metabolic mechanisms that underlie fundamental differences between endothermic and ectothermic species, and its assumptions are checked with sensitivity analysis. Our results provide new support, in agreement with other strong lines of evidence [2], [3], [11], [14], [18]–[20], that endothermy was present in many, if not all, non-avian dinosaurs, especially larger taxa. Endothermy still plausibly was plesiomorphic for Dinosauriformes or even Ornithodira, but this is more ambiguous with our method. This is because these taxa all tend to be small-bodied and thus have relatively low estimated locomotor costs, close to the ecto/endothermy boundary (or areas of overlapping costs between the two groupings). Endothermy, linked to rapid growth and high locomotor aerobic scope, may have presaged the evolution of advanced ventilatory anatomy and function, providing a critical locomotor advantage for dinosauromorphs, particularly in larger species.

## Materials and Methods

### Dinosauriform Sample

We used recent work linking locomotor anatomy to cost to determine the rate of energy expenditure during locomotion for fourteen species of extinct dinosauriform archosaurs, including *Marasuchus* and the giant flightless bird *Dinornis* (Table 1). We applied two different methods, one simple and one more complex, to do this.

Taxa were chosen for completeness, accessibility, and phylogenetic representation of the lineage from basal archosaurs to extant birds. All basal dinosauriforms were assumed to be bipedal although evidence for bipedalism is more ambiguous for some taxa, particularly *Marasuchus* and *Plateosaurus*. Our sampling is far from complete; inclusion of more basal dinosauromorphs/dinosaurs, non-avian coelurosaurs, and basal birds could be interesting but we feel we have captured the basic diversity in body size and locomotor functional morphology with the chosen taxa. Our results suggest that additional taxa would show results similar to those in our sample with similar mass and locomotor anatomy. Adding more basal dinosauromorphs, for example, would add more values similar to those for *Marasuchus*. It is unclear how species with markedly different anatomy, such as large quadrupedal sauropods, would compare to the taxa in our sample.

### Estimating Locomotor Cost from Limb Length

Our first, simpler method estimated the mass-specific locomotor cost of transport, COT (mlO_2_ kg^−1^ m^−1^), using effective limb length (i.e., hip height); the distance from the hip joint to the ground while standing. In a recent comparison of locomotor cost in 28 terrestrial species, including 11 mammals, 8 birds, and 5 reptiles, effective limb length explained 98% of the variation in observed COT [39]. Moreover, once hip height was accounted for there was no independent effect of body mass on cost, indicating that effective limb length is the primary determinant of the well-documented scaling of locomotor cost with body size [39]. Using reconstructed initial limb postures for the dinosaurs in this analysis (Figure 1; Table 1; [40]), we estimated COT from hip height as COT (J kg^−1^ m^−1^) = 90.284(Hip Height, cm)^−0.77^ following Pontzer [39], and converted to mlO_2_ assuming 1 mlO_2_ = 20.1 Joules. This simple approach does not explicitly consider joint mechanics or muscle anatomy in estimating locomotor cost, effectively assuming that dinosauriforms followed the same scaling relationships for muscle fascicle length, l_fasc_, and joint effective mechanical advantage (EMA) as modern birds and mammals do [29], [39], [41].

We multiplied COT by estimated body mass (Table 1) and walking or running speed in order to calculate the whole-body rate of oxygen consumption, VO_2_ (mlO_2_ s^−1^) during locomotion. Note that this approach gives the net rate of locomotor oxygen use, above the baseline rate of resting metabolism. Speeds were tailored to each species' body size using the Froude number [42], where Fr = speed^2^ (hip height·g)^−1^. VO_2_ was calculated at a walk (Fr = 0.25), a slow run (Fr = 0.5), and a moderate run (Froude = 1.0). Although running capacity in the largest theropods remains controversial, biomechanical solutions exist that allow slow to moderate-speed running (below Fr ∼5) [31], [40], [42]–[44] which we focus on here. Speeds and VO_2_ estimates are given in Table 1.

### Estimating Locomotor Cost from Active Muscle Volume

The second, more complex, method we used to reconstruct COT and VO_2_ uses the volume of muscle activated to support and propel the body while walking and running to predict the cost of locomotion. Following previous experimental work [30], [41], [45]–[47] that indicates a strong link between the cost of generating muscular force to support body weight during the stance phase, we developed a model predicting COT (mlO_2_ kg^−1^ m^−1^) from the mass-specific volume of muscle activated, V_musc_ (cm^3^ kg^−1^ m^−1^), to support body weight during walking and running.

Using published data for 10 extant species (Table S1), the volume of muscle activated per unit of ground reaction force (GRF) was estimated as the mean fascicle length of the extensor muscles, l_fasc_, divided by the joint's effective mechanical advantage or EMA [29], [45]–[47]; the posture-dependent ratio of the antigravity muscle and GRF moment arms (r and R, respectively) (Figure 1). Where available, EMA for extant taxa was calculated using force-plate-based measurements of R. For other extant species, EMA was calculated using a free-body diagram of a supportive hindlimb at mid-stance, including segmental gravitational (but not inertial; negligible at midstance) moments [48]. The poses input for these modeled taxa were based upon experimental data for running animals at mid-stance [48].

Where possible, modeled poses were updated with more recent kinematic data. For example, we used more accurate poses for quadrupedal (walking) alligators [49] as well as running ostriches [50], but note that these updated poses did not change our results drastically when compared to prior reconstructions [48] (see Text S1). Also note that the alligator pose used in [48] was bipedal and not identical to the quadrupedal pose used here. Additionally, predicting total muscle volumes solely from hindlimb data for the extant quadrupeds simply assumes that the fore- and hindlimbs are acting with similar mechanical advantage, activating similar volumes of muscle to produce one Newton of GRF. This assumption is supported by force-plate studies in other quadrupeds (dogs [45] and quadrupedal chimpanzees [46]).

Using EMA and cadaver-based estimates of fascicle length, and assuming an isometric muscle stress of 200 kNm^−2^ [46] (plausible variation of this parameter, ±50%, does not affect our ultimate results), we then estimated the volume of muscle needed to produce one Newton of GRF during locomotion. The volume per 1N of GRF at each joint (hip, knee, and ankle) was summed, and then multiplied by g/step length [41] to give the total mass-specific muscle volume, V_musc_, activated per meter travelled [30], [44]–[47]. In our validation test of this model, V_musc_ predicted 98% of the variation in net (i.e., with resting costs removed) mass-specific COT (r^2^ = 0.98, df = 9, p<0.001; Figure S1a), indicating this method reliably predicts locomotor cost across a range of terrestrial vertebrates. This strong correlation remained even when the smallest species, Bobwhite quail, was removed (r^2^ = 0.93, df = 8, p<0.001, Figure S1b). Notably, estimates of V_musc_ from both force-plate studies and from inverse dynamic models fit the V_musc_/COT trendline equally well (Figure S1). Note that degrees of freedom reflect the number of species.

We then applied this validated model to the extinct dinosauriforms in our dataset in order to predict locomotor metabolic rate for these extinct bipeds. V_musc_ for each dinosaur species was calculated as for extant species, using published reconstructions of EMA and l_fasc_ [40], [48]; as in our extant sample, active muscle volume for the metatarsophalangeal joint in our dinosaur sample was excluded (see [40], [48] for discussion). Non-avian dinosaur step lengths were estimated from the ratio of step length to hip height in modern birds (step length = 1.1hip height, ordinary least squares (OLS) regression: r^2^ = 0.87, n = 6, p<0.01). We converted V_musc_ to COT using the OLS equation from our model, as COT = 0.0029 V_musc_+0.0598 (r^2^ = 0.93, df = 8, p<0.001; Figure S1B). As with hip height based estimates of cost (above), we then multiplied COT by estimated body mass and speed to give the whole-body rate of oxygen consumption, VO_2_ (mlO_2_ s^−1^) during locomotion.

### Comparing VO_2_ for Dinosaurs to VO_2_max in Endotherms and Ectotherms

VO_2_ for each dinosauriform was then compared to VO_2_max for extant endotherms and ectotherms. VO_2_max data used in our comparative sample were from measurements reported explicitly as VO_2_max, from the maximum reported aerobic power elicited during treadmill exercise studies, or, for three large varanid lizards, estimated as five times the field metabolic rate [51] measured in active, free-ranging animals. The use of maximum reported aerobic power from exercise studies will tend to underestimate true VO_2_max. While this will have the effect of depressing the VO2max-Body Mass trendline (see below), this effect is very small for the ectotherm group in our sample, since most (89%) of ectotherm measurements are explicit measurements of VO_2_max. The effect on the endotherm trendline is likely somewhat larger; however, the critical comparison for our dinosauriform taxa is to the ectotherm range.

For comparison with net locomotor aerobic power (mlO_2_/s) predicted by our model, we subtracted resting metabolic rate (RMR, Watts), estimated from body mass (kg) using published regressions (mammals [52]: RMR = 3.40mass^0.75^; birds [53]: RMR = 3.79mass^0.72^; reptiles [54]: RMR = 0.69mass^0.82^; all converted to mlO_2_ assuming 1 mlO_2_ = 20.1 Joules, from these VO_2_max measurements, and then plotted them against body mass. All measurements for ectotherms are from body temperatures of 30° to 40°C; when multiple measurements were reported, the measurement in the warmest environment (which typically is the highest VO_2_max value) was used (see Table S2). As reported previously [1], VO_2_max in our sample of endotherms (n = 62 birds and mammals) was an order of magnitude higher than for ectotherms (n = 37 reptiles) of similar body mass (endotherm OLS trendline: VO_2_max = 1.23Mass^0.93^, ectotherm: VO_2_max = 0.16Mass^0.85^, Figure 2).

Next, VO_2_max and body mass were log10 transformed, and compared using OLS in order to calculate the standard error of estimate (SEE) for this relationship. The 95% confidence ranges for ectotherm and endotherm VO_2_max were then calculated as ±2SEE from their respective OLS trendlines. Due to the relatively large sample sizes for endotherms and ectotherms, removal of the largest and smallest taxa from this analysis had negligible effect on the confidence intervals, and did not affect the outcome of our dinosaur comparisons. Similarly, removal of ectothermic taxa for which VO_2_max was estimated (Table S2) did not affect overall results in identifying dinosauriforms as endotherms.

To test this approach for distinguishing ectotherms and endotherms, we calculated V_musc_ and COT as above for three ectothermic and three endothermic species (Table S3). Our approach correctly placed moderate running (Fr = 1.0) for two extant birds, and all locomotion for the extinct moa, in the endotherm range. Similarly, locomotor costs for the bipedal basilisk lizard, and for slow running in quadrupedal iguanas and alligators, were correctly placed in the ectotherm range (Figure S2).

The results of our sensitivity analyses for our models of dinosauriform anatomy, posture, and locomotor cost are outlined above, and are described in the Supporting Information (Text S1).

### Phylogenetic Analysis: When Did Endothermic-Level Locomotor Costs Evolve

To test whether endothermy predated advanced lung structure in Saurischia, we mapped our quantitative data for our hip height and V_musc_-based estimates of locomotor cost onto a consensus phylogeny (Figure 3) of Archosauria, using Mesquite 2.6 [55] and coding the data as qualitative character states (see below). The phylogeny represents an informal “consensus” tree for Archosauria [56]–[68]. Alternative placements for taxa such as *Dilophosaurus* (i.e. moved to sister taxon with *Coelophysis* as [62], [68] rather than the initial position based on [64]) have minimal effects on the results; the positions of other taxa are generally uncontroversial. The data from Table 1 were used to code taxa into four character states of two characters: one from the conservative VO_2_ estimates of locomotor cost for slow walking, and one from the generally higher hip height estimates of locomotor cost. Character states were: (0) known ectotherms, (1) uncertain metabolic status (i.e. locomotor cost estimates within ectotherm 95% CIs), (2) likely endotherms (i.e. locomotor cost estimates above ectotherm 95% CIs) and (3) known endotherms. Parsimony-based character optimization was then implemented in Mesquite to trace the evolution of these characters in Figure 3.

## Supporting Information

Text S1 Explanation of methods and sensitivity analysis.
(0.05 MB DOC)

Table S1 Anatomical measurements, locomotor cost, step length, and active muscle volume for species used to validate the model.
(0.05 MB DOC)

Table S2 Body mass, estimated resting metabolic rate (RMR), and maximum aerobic power (VO_2max_) for extant species. RMR: estimated from mass; see text. Data type: V_O2max_, studies explicitly measuring maximum aerobic power; exercise, from highest reported aerobic power in a locomotion study; 5x FMR, five-times the reported field metabolic rate for this species. Temp.: environmental temperature for measurements of ectotherms.
(0.18 MB DOC)

Table S3 Anatomical parameters, mass-specific active muscle volume (V_musc_) and mass-specific cost of transport (COT) estimated for three endothermic and three ectothermic species.
(0.02 MB DOC)

Figure S1 Mass-specific active muscle volume (V_musc_) versus cost of transport for the extant comparative sample. Black circles: V_musc_ data from force-plate trials, gray circles: V_musc_ modeled from free-body diagram analysis [45]–[47]; see Table S1.
(0.17 MB TIF)

Figure S2 Cost of locomotion at Fr 0.25, 0.5, and 1.0 for three ectotherms (Basiliscus, Iguana, and Alligator, blue circles) and three endotherms (Eudromia, Gallus, and Dinornis, red circles). Symbols as in Figure 2a. Data in Table S3.
(1.35 MB TIF)
