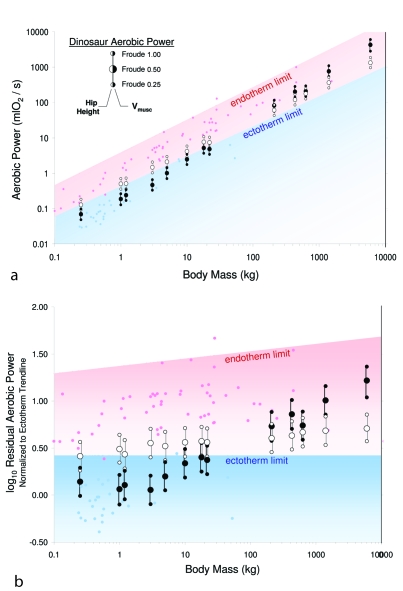# Correction: Biomechanics of Running Indicates Endothermy in Bipedal Dinosaurs

**DOI:** 10.1371/annotation/635e46fc-4be3-4f42-ad5c-ee3a276cd24f

**Published:** 2009-12-01

**Authors:** Herman Pontzer, Vivian Allen, John R. Hutchinson

Figure 2 contains errors. Please view the corrected figure at:

**Figure 2:**