# Supplementary material for: Biomechanics of Running Indicates Endothermy in Bipedal Dinosaurs
Source: PLoS One. 2009 Nov 11;4(11):e7783. doi: 10.1371/journal.pone.0007783 (PMC2772121; doi:10.1371/journal.pone.0007783)
Supplement: Text S1 — Explanation of methods and sensitivity analysis. (0.05 MB DOC) [file pone.0007783.s001.doc]

# Text S1

**SENSITIVITY ANALYSES**

The key unknown parameters in our model are body mass, hip height (which determines COT using the hip height approach and also affects step length and Fr), midstance “external” (GRF and segment gravitational) moment arm (R), antigravity muscle moment arm (r; EMA = r/R), and antigravity muscle fascicle length (l_fasc_). The initial input values are shown in ESM Table 1 below. Here, as an extreme case, we focus our sensitivity analysis on *Tyrannosaurus* because many other studies have already done detailed parameter studies for this taxon [1-4], but our results would apply well to all dinosauriforms in this study.

Of the listed parameters, body mass can be safely excluded from detailed analysis here, as neither approach for estimating COT incorporates body mass, and when COT is multiplied by mass and speed to get COL, the magnitude of the latter value’s change (along the y-axis of Fig. 2) offsets the effect of altered body mass (along the x-axis of Fig. 2). This follows directly from the scaling of VO_2max_ in the extant taxa (see below), which lies close to mass^1.0^. For example there is no appreciable change in where the V_musc_ based estimate for *Tyrannosaurus* plots when given a body mass of 6,000kg (490% the upper bound for ectotherms at Fr=0.50) versus 8400kg (520% the upper bound for ectotherms) (minimal and maximal mass estimates from Hutchinson et al.[3]).

**HIP HEIGHT BASED ESTIMATES OF COST**

*a) Limb Posture and the Scaling of Mechanical Advantage*

Limb pose is one of the most crucial unknowns for dinosaur biomechanics and cannot safely be ignored ([1,4]), because here it affects hip height as well as R (and to a lesser degree, r; [2]). Hip height in *Tyrannosaurus* was probably between 2.31 and 2.93m (using extreme 25% or conservative 5% compression of total leg length for mid-stance limb compression [4]); our initial model used an intermediate value of 2.79m that likely is slightly too crouched. A larger hip height would decrease cost estimates based on hip height, but would also increase the absolute speed at a given Froude number. Thus, the maximally extended posture for *Tyrannosaurus* (2.93m) only decreases estimated cost by 1.3% compared to our reported values using the hip height approach (Table 1). Similar changes would be seen in smaller taxa. Further, it should be noted that hip heights we used are already near their maximum plausible values for the smallest dinosauriforms in this analysis, since the postures used are more extended than expected for animals this size (see below).

**ACTIVE MUSCLE VOLUME BASED ESTIMATES OF COST**

*a) Limb Posture and the Scaling of Mechanical Advantage*

Increasing hip height will also decrease cost estimates using the V_musc_ approach by increasing our step length estimates and hence decrease COT. Still, even using a hip height 2.93m to estimate step length for *Tyrannosaurus* only reduces COT by 3.2% when using the V_musc_ approach. In contrast, a more crouched limb at 2.31m hip height increases COT by 13.6%; such poses are unreasonable for *Tyrannosaurus* but not necessarily so for smaller dinosauriforms [4].

Importantly, as all of our extinct dinosauriform models used the same limb pose [horizontal trunk, 50° included hip angle (trunk-thigh), 110° included knee angle (thigh-shank), 140° degree included ankle angle (shank-foot)], this surely caused a bias to underestimate our V_musc_ based COT values for smaller taxa, as they should have had more crouched poses than the larger taxa. Reconstructed EMA for the limb joints of the extinct taxa barely scales with positive allometry (mass^0.05^, [2]), yet it scales with marked positive allometry (mass^0.26^) in extant mammals [5] and presumably extant dinosauriforms [6]. This is almost certainly not an accurate reflection of real poses in extinct dinosaurs, as their limb bones scaled similarly to mammals [7-9] that show strong positive allometry of EMA [5]. This supports the inference that similar EMA scaling influenced their bone scaling. Resolving this question of EMA scaling more precisely, however, will take more complex dynamic models that are beyond the scope of this study, and will perpetually involve considerable uncertainty.

Reconstructed EMA has no effect on our hip height based estimates of cost since joint postures are not considered in this approach. Thus, our estimates of COT based on hip height scale with dinosauriform body mass^-0.26^, similar to the mass^-0.32^ relationship seen in modern taxa [10]. However, the effect of our EMA scaling regime on our V_musc_ based estimates is evident in the scaling of COT in our extinct dinosaurs, which scales with mass^-0.05^. If EMA scaled in extinct dinosauriforms as mass^0.30^ this would rectify this discrepancy, but that possibility cannot be demonstrated here.

It is plausible that the smallest dinosauriforms in our sample were slightly more crouched, and thus had higher costs, than our reconstructed poses show. Yet it is unlikely that the largest theropod dinosaurs were as columnar as suggested by the hip height-based approach, since the largest theropods would require hyperextended hip and knee joints, and near-vertical orientation of the trunk (see below), to achieve the high EMA’s implicated by the scaling of EMA in modern taxa. In other words, it is unrealistic to infer that EMA could scale positively at a constant slope all the way to the largest theropods; the slope must decline or plateau at some unknown size. Overall, our estimates of EMA affect locomotor cost, but using an EMA scaling regime drawn from extant taxa does not weaken our conclusions, and supports the inference that the primary bias in our reconstructions is underestimating COL for the smallest dinosauriforms. This is why we have inferred that even smaller dinosauriforms probably had locomotor power requirements which exceed modern ectothermic capabilities

More vertical orientations of the axial column in extinct dinosauriforms might seem to be a viable mechanical strategy [11] for reducing at least hip moments and V_musc_, lowering locomotor cost, as here we have assumed a conventional near-horizontal body posture. Yet such vertical trunk orientations are strongly contradicted by anatomical evidence [12], and likely centre of mass positions in dinosaurs included not only a cranial but also a ventral displacement from the hips, which would reduce or even worsen hip moments if the vertebral column was elevated above horizontal [3]. Finally, our center of mass sensitivity analysis (see below) shows that even if the center of mass is coincident with the hip joint in the sagittal plane, and no hip muscles were activated, it would not change our results immensely or invalidate our conclusions. Hence this strategy can be safely excluded.

As the center of mass lay well in front of and below the hips in all dinosaurs (e.g., [3,4,13,14]), including extant forms, the most parsimonious assumption is that at mid-stance the GRF vector would be vertical through the body center of mass, incurring a marked hip flexor moment, not directed through the hip joint as in humans and some quadrupeds. Although the human/quadruped strategy might seem more mechanically sensible in terms of minimizing muscle activity, it is not the strategy used by extant birds and considering the anatomical and geometric evidence the avian strategy is at least common to all dinosaurs. This is reinforced by optimization-based simulations that found qualitatively similar near-horizontal poses in extinct theropods [15].

*b) Step Length, Muscle Moment Arm (r), and GRF Moment Arm (R)*

Our usage of bird step length-hip height relationships (step length = 1.1 hip height, OLS: r^2^=0.87, n=6, p<0.01) to calculate our extinct dinosauriform step lengths introduces some error into our V_musc_ based estimates of cost, as extant avian and extinct dinosaur limb kinematics surely differed and also evolved over time (e.g., [13,16]). Yet our approach should overestimate step length at least for larger dinosaurs (>65 kg), as limb excursion angles decrease with mass in extant theropods [6], so animals beyond the size range of our extant sample should have had even smaller excursion angles, and therefore even shorter steps for a given hip height, than predicted. Hence this is another bias favoring our conclusions—if step length was only 75% of our estimated value, then our COT estimates would be 22% higher.

A more crouched limb also increases COT estimates by increasing R (i.e., mainly increasing GRF flexor moment arms about joints, requiring greater active muscle volumes). This parameter R is extremely sensitive to joint orientation and it is difficult to search all potential poses [4]. It is also very sensitive to the location of the segmental centers of mass, particularly for the trunk. However considering the models of Hutchinson [1] (poses) and Hutchinson et al. [3] (center of mass), in a worst case scenario an error of ~50% is expected for over- or under- estimation of R. If the unlikely case of all three major joints having errors of 50% occurred, this would cause over-/underestimates of ~30% for COT. For example, when active muscle volume at the hip is subtracted from estimated V_musc_, as would occur if the center of mass was coincident with the hip joint in the sagittal plane, estimated COT is decreased by only ~30% for all species.

Muscle moment arms (r) were probably overestimated by Hutchinson [1], which biases our analysis toward lower COT and hence more ectothermic values, so this is not a concern for our conclusions. More realistic, smaller r values would bolster our conclusions, but would require complex 3D musculoskeletal models [2] to refine them and allow pose-dependent r values. For example, if we enter favored r values for *Tyrannosaurus* from Hutchinson et al. [2], our COT estimates would be 12% higher.

*c) Fascicle Length*

Fascicle length l_fasc_ is somewhat more problematic in that the range of potential values for dinosauriforms is very difficult to bound. As l_fasc_ was normalized by segment length for extant taxa and then multiplied by segment length for each extant taxon [1,17], dinosauriforms in the size range of the extant taxa used (~0.4-65kg; *Compsognathus*, *Coelophysis*, and *Velociraptor*, perhaps extending to 0.25kg *Archaeopteryx* and the 210kg juvenile *Gorgosaurus*) may have more reliable values, as dramatic size-related (scaling) specializations were presumably absent. Hence here we focus on fascicle lengths for larger theropods, which at least can be bounded. Their absolute values should have been appreciably larger than in the largest extant taxa for which good data exist (e.g., 65kg ostrich l_fasc_ values for hip, knee, and ankle of 0.135, 0.097, and 0.064m [17]; generally larger values are evident in ~500kg horses, e.g., 0.18, 0.11, and 0.019m respectively [18]), because fascicle length scales variably, but at least increases with size in extant clades [19,20].

The l_fasc_ input values (Table S1) can be treated as upper-end values, even though they may not be long enough (e.g., 1). If a factor of 2x overestimate is used to approximate the lower bound for these fascicle length estimates in *Tyrannosaurus* (0.425, 0.20, and 0.13m for hip, knee, and ankle l_fasc_ values), which is generous considering those published data for extant taxa (body masses <600 kg vs. >6000kg for *Tyrannosaurus*), our COT calculations would be overestimated by 33%. Note that such fascicle lengths are substantially shorter than comparable muscles in even small elephants, or estimations from avian/mammalian muscle scaling data ([19,20]; JR Hutchinson et al., unpubl. data), so this requires extreme speculation.

**SENSITIVITY ANALYSIS SUMMARY**

Taking all of these sources of error into consideration, in a worst-case scenario our estimates of locomotor costs in dinosauriforms would be overestimated by ~65%. However these COT values could also be underestimated by ~50%, considering the same parameters. This can more conclusively be resolved with more complex methods including analyses of joint orientation, muscle moment arms, scaling of muscle dimensions in larger extant taxa (including large reptiles), and body center of mass. Yet our initial estimates here, with total maximal errors of 50-65%, seem to be reasonable approximations that are not strongly biased toward favoring our conclusions, and in many ways are conservatively biased against our conclusions. It would take errors of 500 – 1500% to bring our dinosauriform estimates (for Fr 0.5, the ideal comparison point) firmly into the range of ectotherm locomotor costs, especially for larger taxa.

REFERENCES (also for Table S2)

1. Hutchinson JR (2004b) Biomechanical modeling and sensitivity analysis of bipedal running ability. II. Extinct taxa. J Morphol 262: 441-461.
2. Hutchinson JR, Anderson FC, Blemker S, Delp SL (2005) Analysis of hindlimb muscle moment arms in *Tyrannosaurus rex* using a three-dimensional musculoskeletal computer model. Paleobiol 31: 676-701.
3. Hutchinson JR, Ng-Thow-Hing V, Anderson FC (2007) A 3D interactive method for estimating body segmental parameters in animals: application to the turning and running performance of Tyrannosaurus rex. J Theor Biol 246: 660-680.
4. Gatesy SM, Bäker M, Hutchinson JR (2009) Constraint-based exclusion of limb poses for reconstructing dinosaur locomotion. J Vert Paleontol 29: 535-544.
5. Biewener AA (1989) Scaling body support in mammals: limb posture and muscle mechanics. Science 245: 45-48.
6. Gatesy SM, Biewener AA (1991) Bipedal locomotion: effects of speed, size and limb posture in birds and humans. J Zool 224: 127-147.
7. Gatesy SM (1991b) Hind limb scaling in birds and other theropods: implications for terrestrial locomotion. J Morphol 209: 83-96.
8. Christiansen P (1999) Long bone scaling and limb posture in non-avian theropods: evidence for differential allometry. J Vert Paleont 19: 666–680.
9. Carrano MT (2001) Implications of limb bone scaling, curvature and eccentricity in mammals and non-avian dinosaurs. J Zool 254: 41-55.
10. Taylor CR, Heglund NC, Maloiy GM (1982) Energetics and mechanics of terrestrial locomotion. I. Metabolic energy comsumption as a function of speed and body size in birds and mammals. J Exp Biol 97: 1-21.
11. Carrier DR, Walter RM, Lee DV (2001) Influence of rotational inertia on turning performance of theropod dinosaurs. J Exp Biol 204: 3917-3926.
12. Paul GS (2005) Body and tail posture in theropod dinosaurs. Pp.238-246 in K. Carpenter (ed.), The carnivorous dinosaurs. Indiana Univ Press, Bloomington.
13. Gatesy SM (1990) Caudofemoral musculature and the evolution of theropod locomotion. Paleobiol 16: 170-186.
14. Bates KT, Manning PL, Hodgetts D, Sellers WI (2009) Estimating mass properties of dinosaurs using laser imaging and 3D computer modelling. PLoS One 4(2): e4532.
15. Sellers WI, Manning PL (2007) Estimating dinosaur maximum running speeds using evolutionary robotics. Proc Roy Soc Lond B 274: 2711-2716.
16. Hutchinson JR, Allen V (2009) The evolutionary continuum of limb function from early theropods to birds. Naturwiss 96: 423-448. doi: 10.1007/s00114-008-0488-3
17. Hutchinson JR (2004a) Biomechanical modeling and sensitivity analysis of bipedal running ability. I. Extant taxa. J Morphol 262: 421-440.
18. Payne RC, Hutchinson JR, Robilliard JJ, Smith NC, Wilson AM (2005) Functional specialisation of pelvic limb anatomy in horses (*Equus caballus*). J Anat 206: 557-574.
19. Maloiy GMO, Alexander RM, Njau R, Jayes AS (1979) Allometry of the legs of running birds. J Zool 187: 161-167.
20. Alexander RM, Jayes AS, Maloiy GMO, Wathuta EM (1981) Allometry of the leg muscles of mammals. J Zool 194: 539-552.
21. Fedak MA, Pinshow B, Schmidt-Nielsen K (1974) Energy cost of bipedal running. Am J Physiol 227: 1038-1044.
22. Hammond KA, Chappell MA, Cardullo RA, Lin R-S, Johnsen TS (2000) The mechanistic basis of aerobic performance variation in red junglefowl. J Exp Biol 203: 2053-2064.
23. Weibel ER, Bacigalupe LD, Schmitt B, Hoppeler H (2004) Allometric scaling of maximal metabolic rate in mammals: muscle aerobic capacity as determinant factor. Resp Physiol Neurobiol 140: 115-132.
24. Taylor CR, Schmidt-Nielsen K, Raab JL (1970) Scaling of energetic cost of running to body size in mammals. Am J Physiol 219: 1104-7.
25. Taylor CR, Roundtree V (1973) Temperature regulation in running cheetah: a strategy for sprinters. Am J Physiol. 224: 848-851.
26. Mahoney SA (1980) Cost of locomotion and heat balance during rest and running from 0 to 55 degrees C in a patas monkey. J Appl Physiol 49: 789-800.
27. Taylor CR, Schmidt-Nielsen K, Dmi’el R, Fedak M (1971) Effect of hypothermia on heat balance during running in the African hunting dog. Am J Physiol 220: 823-827.
28. Taylor CR, Maloiy GM, Weibel ER, Langman VA, Kamau JM, et al. (1981) Design of the mammalian respiratory system. IX. Functional and structural limits for oxygen flow. Respir Physiol 44: 25-37.
29. Weibel ER (2000) Symmorphosis: on form and function in shaping life. Cambridge, Mass.: Harvard University Press.
30. Parker KL, Robbins CT, Hanley TA (1984) Energy expenditure for locomotion by mule deer and elk. J Wildl Manage 48: 474 – 488.
31. Vuorimaa T, Virlander R, Kurkilahti P, Vasankari T, Hakkinen K (2006) Acute changes in muscle activation and leg extension performance after different running exercises in elite long distance runners. Eur J Appl Physiol 96: 282-291.
32. Hoyt DF, Taylor CR (1981) Gait and the energetics of locomotion in horses. Nature. 292: 239–240.
33. Weibel ER, Taylor CR, Hoppeler H (1991) The concept of symmorphosis: a testable hypothesis of structure-function relationship. Proc Nat Acad Sci U S A. 88: 10357-61.
34. Langman VA, Roberts TJ, Black J, Maloiy GM, Heglund NC, et al. (1995) Moving cheaply: energetics of walking in the African elephant. J Exp Biol 16: 141-144.
35. Bennett AF (1982) The energetics of repitilian activity. In: Biology of the Reptilia, Volume 13. Ed: Carl Gans. New York: Academic Press, pp. 155-199.
36. Clemente C, Thompson GG, Withers PC, Lloyd DG (2004) Kinematics, maximal metabolic rate, sprint and endurance for a slow-moving lizard, the thorny devil (*Moloch horridus*), Austral J Zool 52: 487-503.
37. John-Adler HB, Garland T, Bennett AF (1986) Locomotor capacities, oxygen consumption, and the cost of locomotion of the shingle-back lizard (*Trachydosaurus rugosus*). Physiol Zool 59: 523 – 531.
38. Wang T, Carrier DR, Hicks JW (1997) Ventilation and gas exchange in lizards during treadmill exercise. J Exp Biol 200: 2629-39.
39. Hartzler LK, Munns SL, Bennett AF, Hicks JW (2006) Recovery from an activity-induced metabolic acidosis in the American alligator, *Alligator mississippiensis*. Comp Biochem Physiol A 143: 368-74.
40. Conley KE, Christian KA, Hoppeler H, Weibel ER (1995) Heart mitochondrial properties and aerobic capacity are similarly related in a mammal and a reptile. J Exp Biol 198: 739-746
41. Beck DD, Dohm MR, Garland T Jr., Ramírez-Bautista A, Lowe CH (1995) Locomotor performance and activity energetics of helodermatid lizards. Copeia 3: 577-585.
42. Secor SM, Hicks JW, Bennett AF (2000) Ventilatory and cardiovascular responses of a python (*Python molurus*) to exercise and digestion. J Exp Biol 203: 2447-2454.
43. Nagy KA, Girard IA, Brown TK (1999) Energetics of free-ranging mammals, reptiles, and birds. Ann Rev Nutr 19: 247 – 277.
44. Benedict FG (1932) The physiology of large reptiles: with special reference to the heat production of snakes, tortoises, lizards and alligators. Washington: Carnegie Institution of Washington.
