# Supplementary material for: Biomechanics of Running Indicates Endothermy in Bipedal Dinosaurs
Source: PLoS One. 2009 Nov 11;4(11):e7783. doi: 10.1371/journal.pone.0007783 (PMC2772121; doi:10.1371/journal.pone.0007783)
Supplement: Table S1 — Anatomical measurements, locomotor cost, step length, and active muscle volume for species used to validate the model. (0.05 MB DOC) [file pone.0007783.s002.doc]

| **Table S1.** | | | | |  |  |  |  |  |
| --- | --- | --- | --- | --- | --- | --- | --- | --- | --- |
|  | **Mass** | **Hip Height** | **Cost of Transport** | | **L_step_** | | **V_musc_** | | |
|  |
| **Species** | kg | cm | mlO_2_ /(kg m) | source | m | source | cm^3^/(kg m) | source | |
| Bobwhite quail | 0.13 | 10 | 1.05 | [1] | 0.11 | [1] | 416.5 | forceplate | [2] |
| Guinea Fowl | 1.3 | 20 | 0.42 | [1] | 0.38 | [1] | 125.3 | forceplate | [2] |
| Dog | 4.5 | 23 | 0.29 | [1] | 0.27 | [1] | 69.7 | forceplate | [2] |
| Turkey | 5.3 | 36 | 0.31 | [1] | 0.55 | [1] | 85.9 | forceplate | [2] |
| Rhea | 19.9 | 82 | 0.21 | [1] | 0.76 | [1] | 62.3 | forceplate | [2] |
| Emu | 40.1 | 82 | 0.18 | [1] | 0.93 | [1] | 51.2 | forceplate | [2] |
| Chimpanzee (quadrupedal) | 49.8 | 50 | 0.17 | [3] | 0.90 | [3] | 43.7 | forceplate | [2] |
| Chimpanzee (bipedal) | 49.8 | 50 | 0.25 | [3] | 0.64 | [3] | 58.2 | forceplate | [2] |
| Human (running) | 69 | 90 | 0.19 | Pontzer unpub data | 0.82 | [1] | 32.4 | forceplate | [2] |
| Human (walking) | 69.3 | 92 | 0.10 | [3] | 0.83 | [3] | 11.4 | forceplate | [2] |
| Turkey | 3.7 | 28 | 0.31 | [1] | 0.55 | [1] | 87.7 | modeled | [4] |
| Red kangaroo | 6.6 | 92 | 0.34 | [5] | 0.54 | [6]^a^ | 80.8 | modeled | [4] |
| Emu | 27.2 | 66 | 0.18 | [1] | 0.93 | [1] | 46.6 | modeled | [4] |
| Ostrich | 65.3 | 85 | 0.11 | [7] | 0.94 | estimate^b^ | 20.3 | modeled | [4] |
| Human (running) | 71 | 86 | 0.18 | [2] | 0.82 | [2] | 40.0 | modeled | [4] |
|  |  |  |  |  |  |  |  |  |  |
| a. Adapted from data for red kangaroos, scaled isometrically to 4.9kg, same mass as data for COT; reference: Kram R, Dawson TJ (1998) Energetics and biomechanics of locomotion by red kangaroos (*Macropus rufus*). Comp Biochem Physiol B 120:41-49. | | | | | | |  |  |  |
| b. Estimated using L_step_/Hip Height ratio of other birds in sample | | | | | | |  |  |  |

1. **Roberts TJ, Chen MS, Taylor CR (1998a) Energetics of bipedal running: II. Metabolic cost of generating force. J Exp Biol 201: 2745-2751.**
2. **Pontzer H, Raichlen DA, Sockol MD (2009) The metabolic cost of walking in humans, chimpanzees, and early hominins. J H Evol 56: 43-54.**
3. **Sockol MD, Raichlen DA, Pontzer H (2007) Chimpanzee locomotor energetics and the origin of human bipedalism. Proc Nat Acad Sci 30: 12265-12269.**
4. **Hutchinson JR (2004a) Biomechanical modeling and sensitivity analysis of bipedal running ability. I. Extant taxa. J Morphol 262: 421-440.**
5. **Baudinette RV, Snyder GK, Frappell PB (1992) Energetic cost of locomotion in the tammar wallaby. Am J Physiol Regul Integr Comp Physiol 262: 771-778**
6. **Kram R, Dawson TJ (1998) Energetics and biomechanics of locomotion by red kangaroos (Macropus rufus). Comp. Biochem. Physiol. B 120:41-49.**
7. **Taylor CR, Heglund NC, Maloiy GM (1982) Energetics and mechanics of terrestrial locomotion. I. Metabolic energy comsumption as a function of speed and body size in birds and mammals. J. Exp. Biol. 97:1-21.**
