# Supplementary material for: Biomechanics of Running Indicates Endothermy in Bipedal Dinosaurs
Source: PLoS One. 2009 Nov 11;4(11):e7783. doi: 10.1371/journal.pone.0007783 (PMC2772121; doi:10.1371/journal.pone.0007783)
Supplement: Table S2 — Body mass, estimated resting metabolic rate (RMR), and maximum aerobic power (VO2max) for extant species. RMR: estimated from mass; see text. Data type: VO2max, studies explicitly measuring maximum aerobic power; exercise, from highest reported aerobic power in a locomotion study; 5x FMR, five-times the reported field metabolic rate for this species. Temp.: environmental temperature for measurements of ectotherms. (0.18 MB DOC) [file pone.0007783.s003.doc]

| **Table S2.** | | | | | | | | |
| --- | --- | --- | --- | --- | --- | --- | --- | --- |
|  |  |  |  | **Mass** | **RMR** | **VO_2_max** | **Notes** | |
| **Group** | **Category** | **Common Name** | **Species** | kg | mlO_2_/s | mlO_2_/ s | Source | Data type |
| Bird | endotherm | Painted quail | *E. chinensis* | 0.042 | 0.02 | 0.06 | Fedak et al 1974 | exercise |
| Bird | endotherm | Bobwhite quail | *Colinus virginianus* | 0.19 | 0.05 | 0.20 | Fedak et al 1974 | exercise |
| Bird | endotherm | Chukar | *Anser graeca* | 0.49 | 0.10 | 0.57 | Fedak et al 1974 | exercise |
| Bird | endotherm | Guinea fowl | *Numida meleagris* | 1.2 | 0.19 | 1.42 | Fedak et al 1974 | exercise |
| Bird | endotherm | Goose | *Anser anser* | 3.8 | 0.46 | 2.92 | Fedak et al 1974 | exercise |
| Bird | endotherm | Turkey | *Meleagris gallopavo* | 4.3 | 0.51 | 5.54 | Fedak et al 1974 | exercise |
| Bird | endotherm | Rhea | *Rhea americana* | 22 | 1.72 | 28.41 | Fedak et al 1974 | exercise |
| Bird | endotherm | Junglefowl | *Gallus gallus* | 1.1 | 0.18 | 1.58 | Hammond et al 2003 | VO_2_ max |
| Mammal | endotherm | Pygmy mouse | *Baiomys taylori* | 0.007 | 0.004 | 0.03 | Weibel et al 2004 | VO_2_ max |
| Mammal | endotherm | Woodmouse | *Apodemus sylvaticus* | 0.020 | 0.009 | 0.09 | Weibel et al 2004 | VO_2_ max |
| Mammal | endotherm | White mouse | *Mus musculus* | 0.021 | 0.009 | 0.04 | Taylor et al 1970 | exercise |
| Mammal | endotherm | Deer mouse | *Peromyscus maniculatus* | 0.022 | 0.010 | 0.08 | Weibel et al 2004 | VO_2_ max |
| Mammal | endotherm | Merriams Kangaroo rat | *Dipodymus merriami* | 0.041 | 0.015 | 0.05 | Taylor et al 1970 | exercise |
| Mammal | endotherm | Flying squirrel | *Glaucomys volans* | 0.063 | 0.021 | 0.08 | Taylor et al 1982 | exercise |
| Mammal | endotherm | Chipmunk | *Tamais striatus* | 0.090 | 0.028 | 0.24 | Weibel et al 2004 | VO_2_ max |
| Mammal | endotherm | Bannertailed Kangaroo Rat | *Dipodymus spectabilis* | 0.10 | 0.030 | 0.11 | Taylor et al 1970 | exercise |
| Mammal | endotherm | Tree shrew | *Tupia glis* | 0.12 | 0.035 | 0.14 | Taylor et al 1982 | exercise |
| Mammal | endotherm | Mole rat | *Spalax ehrenbergi* | 0.14 | 0.038 | 0.39 | Weibel et al 2004 | VO_2_ max |
| Mammal | endotherm | Ground Squirrel | *C. tereticaudus* | 0.24 | 0.057 | 0.20 | Taylor et al 1970 | exercise |
| Mammal | endotherm | Bushbaby | *Galago senegalensis* | 0.24 | 0.058 | 0.28 | Taylor et al 1982 | exercise |
| Mammal | endotherm | White Rat | *Rattus norvegicus* | 0.38 | 0.083 | 0.40 | Taylor et al 1970 | exercise |
| Mammal | endotherm | Ferret | *Mustela nigripes* | 0.54 | 0.107 | 0.80 | Taylor et al 1982 | exercise |
| Mammal | endotherm | Dwarf mongoose | *Helogale parvula* | 0.58 | 0.113 | 1.12 | Taylor et al 1982 | exercise |
| Mammal | endotherm | Guinea pig | *Cavia procellus* | 0.58 | 0.113 | 0.36 | Weibel et al 2004 | VO_2_ max |
| Mammal | endotherm | Banded mongoose | *Mungos mungo* | 1.2 | 0.188 | 2.08 | Taylor et al 1982 | exercise |
| Mammal | endotherm | Genet cat | *Genetta tigrina* | 1.5 | 0.224 | 2.65 | Taylor et al 1982 | exercise |
| Mammal | endotherm | Dog | *Canis familiaris* | 2.6 | 0.346 | 3.05 | Taylor et al 1970 | exercise |
| Mammal | endotherm | Spring hare | *Pedetes capensis* | 3.0 | 0.386 | 4.86 | Weibel et al 2004 | VO_2_ max |
| Mammal | endotherm | Agouti | *Agouti paca* | 3.2 | 0.407 | 5.47 | Weibel et al 2004 | VO_2_ max |
| Mammal | endotherm | Capuchin | *Cebus capucinus* | 3.3 | 0.418 | 4.30 | Taylor & Rowntree, 1973 | exercise |
| Mammal | endotherm | Suni | *Neotragus moschatus* | 3.5 | 0.433 | 5.71 | Taylor et al 1982 | exercise |
| Mammal | endotherm | Patas monkey | *Erythrocebus patas* | 3.8 | 0.460 | 6.38 | Mahoney 1980 | exercise |
| Mammal | endotherm | Cat | *Felis cattus* | 3.9 | 0.469 | 2.69 | Taylor et al 1982 | exercise |
| Mammal | endotherm | Dik Dik | *Madoqua kirkii* | 4.4 | 0.510 | 3.38 | Taylor et al 1982 | exercise |
| Mammal | endotherm | Dog | *Canis familiaris* | 4.4 | 0.510 | 5.97 | Taylor et al 1982 | exercise |
| Mammal | endotherm | Fox | *Alopex lagopus* | 4.5 | 0.523 | 14.96 | Weibel et al 2004 | VO_2_ max |
| Mammal | endotherm | Stumptail macaque | *Macaca speciosa* | 5.1 | 0.574 | 8.19 | Taylor et al 1982 | exercise |
| Mammal | endotherm | Hamadryas baboon | *Papio hamadryas* | 8.5 | 0.842 | 12.32 | Taylor et al 1982 | exercise |
| Mammal | endotherm | Cape Hunting Dog | *Lycaon pictus* | 8.8 | 0.861 | 13.69 | Taylor et al 1971 | exercise |
| Mammal | endotherm | Grant's gazelle | *Gazella granti* | 11.2 | 1.04 | 9.97 | Taylor et al 1981 | exercise |
| Mammal | endotherm | Coyote | *Canis latrans* | 12.4 | 1.12 | 38.06 | Weibel et al 2004 | VO_2_ max |
| Mammal | endotherm | Chimpanzee | *Pan troglodytes* | 17.5 | 1.45 | 13.63 | Taylor & Rowntree, 1973 | exercise |
| Mammal | endotherm | Pig | *Sus scrofa* | 18.5 | 1.51 | 28.86 | Weibel et al 2004 | VO_2_ max |
| Mammal | endotherm | African Goat | *Capra hircus* | 20.0 | 1.60 | 15.73 | Taylor et al 1982 | exercise |
| Mammal | endotherm | Fat tail sheep | *Ovis aries* | 23.0 | 1.78 | 18.23 | Taylor et al 1982 | exercise |
| Mammal | endotherm | Wolf | *Canis lupus* | 23.1 | 1.78 | 35.79 | Taylor et al 1982 | exercise |
| Mammal | endotherm | Gazelle | *Gazella gazella* | 24.2 | 1.85 | 28.77 | Taylor et al 1974 | exercise |
| Mammal | endotherm | Goat | *Capra hircus* | 27.2 | 2.01 | 17.45 | Taylor et al 1974 | exercise |
| Mammal | endotherm | Lion | *Panthera leo* | 27.5 | 2.03 | 27.50 | Taylor et al 1981 | exercise |
| Mammal | endotherm | Cheetah | *Acinonyx jubatus* | 28.1 | 2.06 | 18.03 | Taylor et al 1974 | exercise |
| Mammal | endotherm | Dog | *Canis familiaris* | 28.2 | 2.07 | 64.39 | Weibel 2000 | VO_2_ max |
| Mammal | endotherm | Pronghorn | *Antelocapra americana* | 28.4 | 2.08 | 128.75 | Weibel 2000 | VO_2_ max |
| Mammal | endotherm | Elk | *Cervus elaphus* | 42.0 | 2.79 | 46.04 | Parker et al 1984 | exercise |
| Mammal | endotherm | Human | *Homo sapiens* | 65.4 | 3.89 | 80.88 | Vuorimma et al 2006 | VO_2_ max |
| Mammal | endotherm | Wildebeest | *Connaechtes taurinus* | 92.0 | 5.02 | 50.90 | Taylor et al 1982 | exercise |
| Mammal | endotherm | Waterbuck | *Kobus defassa* | 114 | 5.90 | 88.46 | Taylor et al 1982 | exercise |
| Mammal | endotherm | Horse | *Equus callabus* | 140 | 6.88 | 104.66 | Hoyt & Taylor 1981 | exercise |
| Mammal | endotherm | Eland | *Taurotragus oryx* | 213 | 9.43 | 129.11 | Taylor et al 1982 | exercise |
| Mammal | endotherm | Zebu | *Bos indicus* | 254 | 10.76 | 124.95 | Taylor et al 1982 | exercise |
| Mammal | endotherm | Horse | *Equus callabus* | 450 | 16.53 | 1003.50 | Weibel et al 1991 | VO_2_ max |
| Mammal | endotherm | Steer | *Bos taurus* | 450 | 16.53 | 382.50 | Weibel et al 1991 | VO_2_ max |
| Mammal | endotherm | African elephant | *Loxodonta africana* | 1542 | 41.62 | 434.60 | Langman et al 1995 | exercise |

| Table S2 cont’d | | | | |  |  |  | **Mass** | **RMR** | **VO_2_max** | **Notes** | |
| --- | --- | --- | --- | --- | --- | --- | --- | --- | --- | --- | --- | --- |
| **Group** | **Temp.** | | | | **Category** | **Common Name** | **Species** | kg | mlO_2_/s | mlO_2_/ s | VO_2_max source | Data type |
| Reptile | 30 | | | | ectotherm | lizard | *Lacerta spp.* | 0.010 | 0.0008 | 0.0041 | Bennett 1982 | VO_2_ max |
| Reptile | 40 | | | | ectotherm | lizard | *Sceloporus occidentalis* | 0.013 | 0.0010 | 0.0066 | Bennett 1982 | VO_2_ max |
| Reptile | 40 | | | | ectotherm | lizard | *Cnemidophorus tigris* | 0.018 | 0.0013 | 0.0105 | Bennett 1982 | VO_2_ max |
| Reptile | | | | 35 | ectotherm | lizard | *Gerrhonotus multicarinatus* | 0.024 | 0.0016 | 0.0075 | Bennett 1982 | VO_2_ max |
| Reptile | | 35 | | | ectotherm | Thorny devil | *Moloch horridus* | 0.030 | 0.0019 | 0.0083 | Clemente et al 2004 | VO_2_ max |
| Reptile | | | 40 | | ectotherm | lizard | *Dipsosaurus dorsalis* | 0.035 | 0.0022 | 0.024 | Bennett 1982 | VO_2_ max |
| Reptile | 40 | | | | ectotherm | lizard | *Cnemidophorus murinus* | 0.055 | 0.0032 | 0.025 | Bennett 1982 | VO_2_ max |
| Reptile | 40 | | | | ectotherm | lizard | *Amphibolurus barbatus* | 0.239 | 0.0106 | 0.041 | Bennett 1982 | VO_2_ max |
| Reptile | 40 | | | | ectotherm | lizard | *Egernia cunninghami* | 0.257 | 0.0113 | 0.040 | Bennett 1982 | VO_2_ max |
| Reptile | 35 | | | | ectotherm | snake | *Coluber constrictor* | 0.262 | 0.0114 | 0.074 | Bennett 1982 | VO_2_ max |
| Reptile | 35 | | | | ectotherm | snake | *Crotalus viridus* | 0.301 | 0.0128 | 0.044 | Bennett 1982 | VO_2_ max |
| Reptile | 40 | | | | ectotherm | turtle | *Pseudemys scripta* | 0.305 | 0.0130 | 0.087 | Bennett 1982 | VO_2_ max |
| Reptile | 40 | | | | ectotherm | turtle | *Terrapene ornata* | 0.354 | 0.0147 | 0.072 | Bennett 1982 | VO_2_ max |
| Reptile | 40 | | | | ectotherm | snake | *Spalerosophis diadema* | 0.386 | 0.0157 | 0.070 | Bennett 1982 | VO_2_ max |
| Reptile | 40 | | | | ectotherm | lizard | *Trachydosaurus rugosus* | 0.421 | 0.0169 | 0.060 | Bennett 1982 | VO_2_ max |
| Reptile | 35 | | | | ectotherm | Shingleback lizard | *Trachydosaurus rugosus* | 0.432 | 0.0172 | 0.087 | John-Alder 1986 | VO_2_ max |
| Reptile | 35 | | | | ectotherm | Monitor lizard | *Varanus exanthematicus* | 0.460 | 0.0182 | 0.097 | Wang et al. 1997 | exercise |
| Reptile | 30 | | | | ectotherm | Alligator | *Alligator mississippiensis* | 0.479 | 0.0188 | 0.144 | Hartzler et al 2006 | VO_2_ max |
| Reptile | 40 | | | | ectotherm | lizard | *Amblyrhynchus cristatus* | 0.489 | 0.0191 | 0.102 | Bennett 1982 | VO_2_ max |
| Reptile | 40 | | | | ectotherm | lizard | *Tiliqua scincoides* | 0.493 | 0.0192 | 0.055 | Bennett 1982 | VO_2_ max |
| Reptile | 35 | | | | ectotherm | snake | *Pituophis catenifer* | 0.548 | 0.0210 | 0.079 | Bennett 1982 | VO_2_ max |
| Reptile | 40 | | | | ectotherm | lizard | *Physignathus lesueuri* | 0.549 | 0.0210 | 0.069 | Bennett 1982 | VO_2_ max |
| Reptile | 31 | | | | ectotherm | Gila monster | *Heloderma suspectum* | 0.557 | 0.0212 | 0.148 | Beck et al 1995 | VO_2_ max |
| Reptile | 40 | | | | ectotherm | lizard | *Sauromalus hispidus* | 0.574 | 0.0218 | 0.093 | Bennett 1982 | VO_2_ max |
| Reptile | 40 | | | | ectotherm | lizard | *Varanus gouldii* | 0.674 | 0.0248 | 0.189 | Bennett 1982 | VO_2_ max |
| Reptile | 40 | | | | ectotherm | lizard | *Varanus spp.* | 0.714 | 0.0260 | 0.149 | Bennett 1982 | VO_2_ max |
| Reptile | 40 | | | | ectotherm | lizard | *Iguana iguana* | 0.795 | 0.0284 | 0.113 | Bennett 1982 | VO_2_ max |
| Reptile | 35 | | | | ectotherm | lizard | *Varanus exanthematicus* | 1.025 | 0.0350 | 0.359 | Bennett 1982 | VO_2_ max |
| Reptile | 35 | | | | ectotherm | Iguana | *Iguana iguana* | 1.115 | 0.0375 | 0.186 | Wang et al. 1997 | VO_2_ max |
| Reptile | 38 | | | | ectotherm | Cuban iguana | *Cyclura nubila* | 1.180 | 0.0393 | 0.282 | Conley et al.1995 | VO_2_ max |
| Reptile | 31 | | | | ectotherm | Mexican bearded lizard | *Heloderma horridum* | 1.194 | 0.0397 | 0.309 | Beck et al 1995 | VO_2_ max |
| Reptile | 30 | | | | ectotherm | Python | *Python molurus* | 1.38 | 0.0447 | 0.205 | Secor et al 2000 | VO_2_ max |
| Reptile | 30 | | | | ectotherm | turtle | *Chelydra serpentina* | 3.47 | 0.0953 | 0.473 | Bennett 1982 | VO_2_ max |
| Reptile | N/A | | | | ectotherm | varanid | *Varanus benegalensis* | 7.53 | 0.180 | 2.61 | Nagy et al 1999 | 5 x FMR |
| Reptile | N/A | | | | ectotherm | varanid | *Varanus salvator* | 7.70 | 0.183 | 2.32 | Nagy et al 1999 | 5 x FMR |
| Reptile | N/A | | | | ectotherm | Komodo dragon | *Varanus komodensis* | 45.2 | 0.781 | 7.01 | Nagy et al 1999 | 5 x FMR |
| Reptile | 30 | | | | ectotherm | Alligator | *Alligator mississippiensis* | 53.0 | 0.890 | 2.92 | Benedict 1932 | VO_2_ max |
