# Supplementary material for: Biomechanics of Running Indicates Endothermy in Bipedal Dinosaurs
Source: PLoS One. 2009 Nov 11;4(11):e7783. doi: 10.1371/journal.pone.0007783 (PMC2772121; doi:10.1371/journal.pone.0007783)
Supplement: Table S3 — Anatomical parameters, mass-specific active muscle volume (Vmusc) and mass-specific cost of transport (COT) estimated for three endothermic and three ectothermic species. (0.02 MB DOC) [file pone.0007783.s004.doc]

**Table S3.**

|  | **Mass** | **Hip Height** | **L_step_** | **V_musc_** | **COT** |
| --- | --- | --- | --- | --- | --- |
| **Species** | kg | cm | m | cm^3^/m | mlO_2_ /(kg m) |
| *Eudromia* (endotherm) | 0.41 | 14 | 0.15 | 155.6 | 0.51 |
| *Gallus* (endotherm) | 2.89 | 23 | 0.22 | 85.8 | 0.31 |
| *Basiliscus* (ectotherm) | 0.19 | 10 | 0.22 | 54.5 | 0.22 |
| *Iguana* (ectotherm) | 4.04 | 18 | 0.38 | 87.2 | 0.32 |
| *Alligator* (ectotherm) | 5.91 | 17 | 0.60 | 83.4 | 0.30 |
| *Dinornis* (endotherm) | 280 | 131 | 1.44 | 33.0 | 0.16 |
