# Supplementary figures and images for: Biomechanics of Running Indicates Endothermy in Bipedal Dinosaurs
Source: PLoS One. 2009 Nov 11;4(11):e7783. doi: 10.1371/journal.pone.0007783 (PMC2772121; doi:10.1371/journal.pone.0007783)

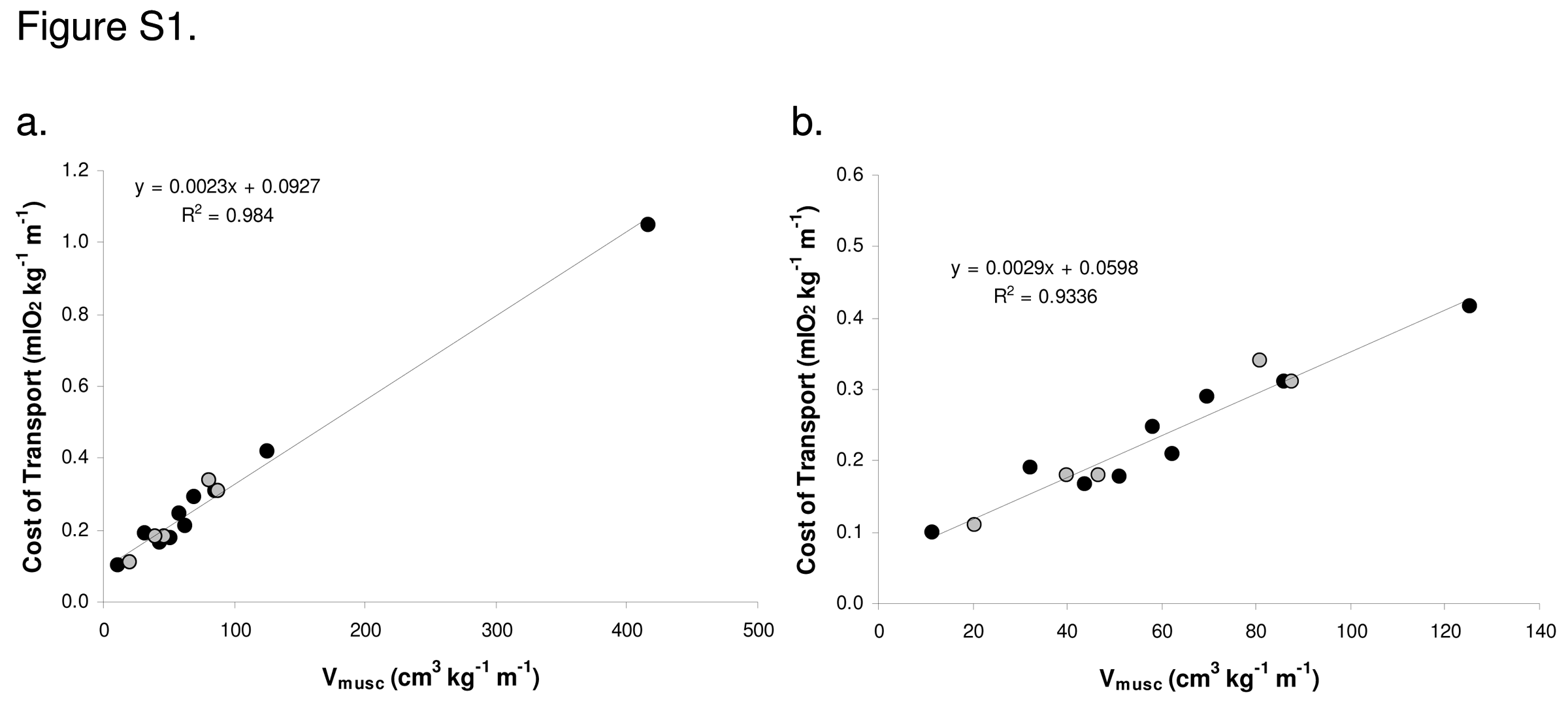

Supplement: Figure S1 — Mass-specific active muscle volume (Vmusc) versus cost of transport for the extant comparative sample. Black circles: Vmusc data from force-plate trials, gray circles: Vmusc modeled from free-body diagram analysis [45]–[47]; see Table S1. (0.17 MB TIF) [file pone.0007783.s005.tif]

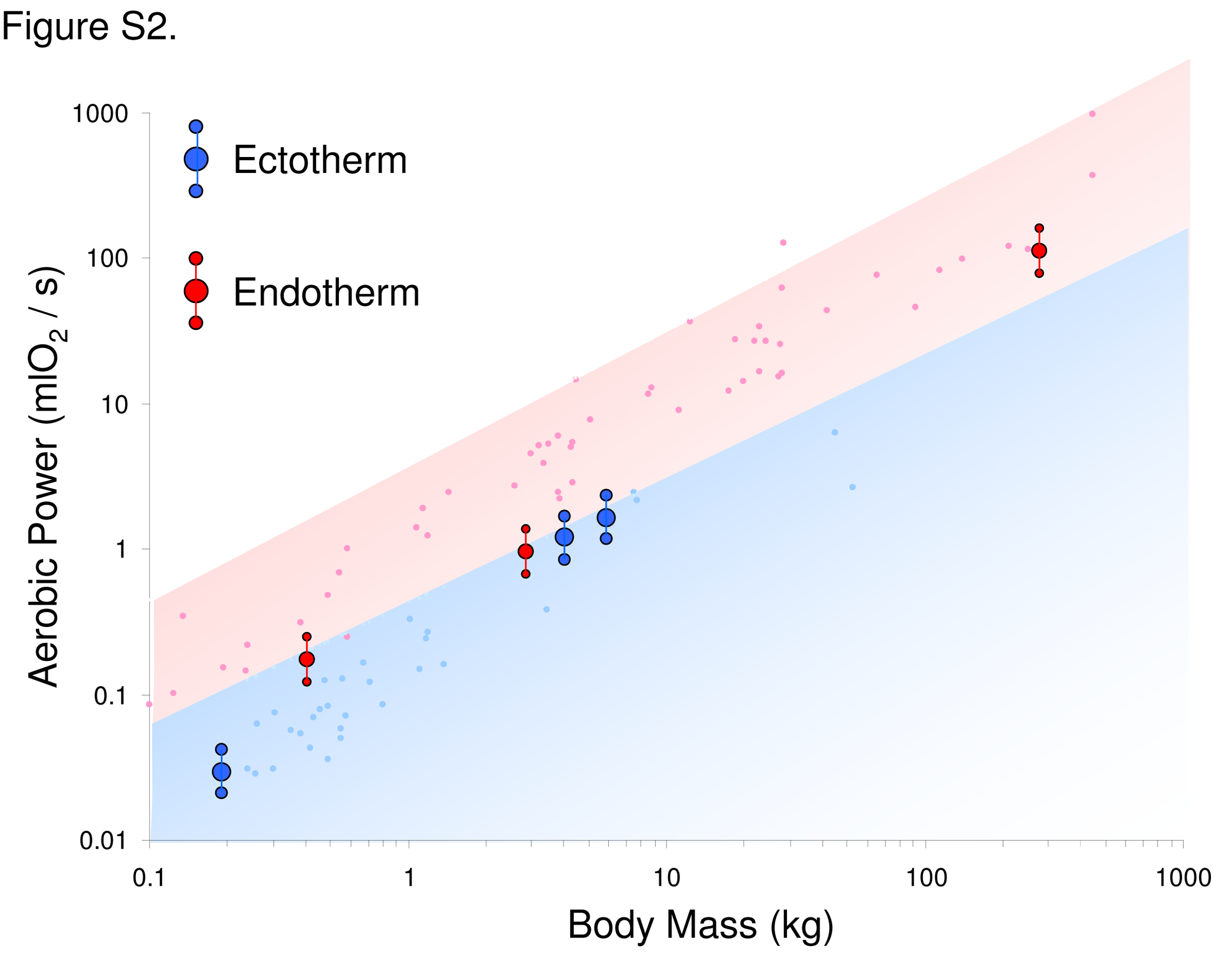

Supplement: Figure S2 — Cost of locomotion at Fr 0.25, 0.5, and 1.0 for three ectotherms (Basiliscus, Iguana, and Alligator, blue circles) and three endotherms (Eudromia, Gallus, and Dinornis, red circles). Symbols as in Figure 2a. Data in Table S3. (1.35 MB TIF) [file pone.0007783.s006.tif]
